# Dynamic Reconfiguration of Functional Topology in Human Brain Networks: From Resting to Task States

**DOI:** 10.1155/2020/8837615

**Published:** 2020-09-08

**Authors:** Wenhai Zhang, Fanggui Tang, Xiaolin Zhou, Hong Li

**Affiliations:** ^1^College of Education Science, Hengyang Normal University, Hengyang 421002, China; ^2^Mental Health Center, Yancheng Institute of Technology, Yancheng 224051, China; ^3^School of Psychology and Cognition, Peking University, Beijing 100871, China; ^4^Institute for Brain and Psychological Sciences, Sichuan Normal University, Chengdu 610066, China

## Abstract

Task demands evoke an intrinsic functional network and flexibly engage multiple distributed networks. However, it is unclear how functional topologies dynamically reconfigure during task performance. Here, we selected the resting- and task-state (emotion and working-memory) functional connectivity data of 81 health subjects from the high-quality HCP data. We used the network-based statistic (NBS) toolbox and the Brain Connectivity Toolbox (BCT) to compute the topological features of functional networks for the resting and task states. Graph-theoretic analysis indicated that under high threshold, a small number of long-distance connections dominated functional networks of emotion and working memory that exhibit distinct long connectivity patterns. Correspondently, task-relevant functional nodes shifted their roles from within-module to between-module: the number of connector hubs (mainly in emotional networks) and kinless hubs (mainly in working-memory networks) increased while provincial hubs disappeared. Moreover, the global properties of assortativity, global efficiency, and transitivity decreased, suggesting that task demands break the intrinsic balance between local and global couplings among brain regions and cause functional networks which tend to be more separated than the resting state. These results characterize dynamic reconfiguration of large-scale distributed networks from resting state to task state and provide evidence for the understanding of the organization principle behind the functional architecture of task-state networks.

## 1. Introduction

Understanding how the brain shapes mind, such as cognition and emotion, ultimately relies on the knowledge of large-scale brain networks [1]. The Human Connectome Project (HCP) used high-quality neuroimaging to map the structural and functional connectivity of the normal human brain [2], which provides new opportunity to understand general topological principles of brain network organization. Graph theory-based connectivity research has shown that a brain network is composed of functionally separate subnetworks or modules [3]. As a complex system, the brain flexibly processes multiple incoming information through interplaying between distributed subsystems [4, 5]. Moreover, the intrinsic functional network during resting state primarily shapes a standard architecture of task-based functional brain organization and is secondarily evoked by task-relevant networks [6]. However, little is known about how the functional topology dynamically reconfigures for task performance.

In graph-theoretic research, a function network is described as a graph with a collection of nodes representing brain regions and edges representing functional interactions in the brain [7, 8]. Nodes are further grouped into module or community with highly connected within-group links and a minimally possible number of between-group links [9]. Human brain networks have complex local and global topological properties (e.g., hub nodes, modules, transitivity measuring functional separation, and global efficiency measuring functional integration) [8]. When task demands change from resting state to task state, metabolic energy is necessarily redistributed to support the reorganized functional architecture [10] and the functional network is dynamically reorganized according to the specific cognitive demands of the task [11]. Correspondently, functional topologies such as connections between/within modules, nodal features, and global features (e.g., transitivity and global efficiency) are reconfigured [12–14]. However, there are still the following three unsolved questions.

First, there is lack of enough evidences to determine how long connections switch from resting state to task states, e.g., emotion and working memory (WM). The functional connectivity refers to some forms of statistical dependency between nodes, and short-distance links are distributed mainly within communities while long-distance links are distributed between communities [15, 16]. During tasks, short connections within communities decrease while long connections between communities increase [6, 17]. A recent meta-analysis indicated that the coactivation networks elicited by a wide range of tasks have more long-range connections [18]. Particularly, the default mode network (DMN) actively contributes to function integration [19]: intra-DMN connectivity decreased while inter-DMN connectivity increased during a 2-back versus a 1-back working memory (WM) task [13]. Moreover, emotion processing (e.g., reappraisal) produces distributed alterations in functional connections involving visual, dorsal attention, frontoparietal, and DMN modules [20]. Long connections between communities are particularly important for brain function because they are responsible for intermodular communication [12, 21]. However, performing statistical testing on connectivity values for large networks suffers from multiple comparison problem so that long-links are easily ignored because of their weak connectivity values [7, 22]. The network-based statistic (NBS) has greater power to detect a whole cluster of regions spanning multiple connections and makes it possible to find a set of connections forming a subnetwork associated with an experimental effect [23]. Here, we used the NBS to further clarify how long connections change during the WM and emotional task versus the resting state.

Second, it is unclear how functional hubs dynamically change their nodal roles during the WM and emotional tasks. Resting-state fMRI research has demonstrated functional hubs distributing in the heteromodal association cortex (e.g., the precuneus, posterior and anterior cingulate gyrus, ventromedial frontal cortex, and inferior parietal regions) [3, 24]. Hubs flexibly process multiple information and rapidly update their connectivity pattern according to task demands [25, 26]. Hub nodes are generally divided into three different roles: provincial hubs with the vast majority of links within their module, connector hubs with many links to most of the other modules, and kinless hubs with links homogeneously distributed among all modules [27]. Finc et al. [13] found that the number of connector hubs increased whereas the number of provincial hubs decreased when the WM task became more demanding. Moreover, task-relevant nodes within auditory, visual, salience, and context community become activated in the WM task while subcortical regions (e.g., amygdala and putamen) take an important role in emotional tasks [20, 26, 28–30]. However, the previous studies ignored the shifting of nonhubs to hubs and rarely mentioned kinless hubs.

Third, it is unclear whether intrinsic functional networks become more integrated or separated during the shift from resting state to task state. A number of structural and resting-state fMRI studies have indicated that brain networks exhibit economical small-world topology [31–33], balancing integration and segregation between brain regions [34, 35]. To satisfy ever changing task demands, the global properties (e.g., clustering and modularity) of brain network organization are responsive to the changing task contexts [12]. Some studies have found that functional networks tend to be of higher global network integration at task state: for example, the performance of cognitive tasks (including WM) is associated with increased global efficiency and less segregation of processing relative to resting state [36, 37]. Other studies have proposed that the global topological properties are largely invariant in order to continually maintaining the balance of efficient local and global processing [38, 39]. Another studies demonstrated that functional networks tend to be highly separated (e.g., negative assortativity coefficients) and exhibit a more random configuration at higher levels of task difficulty (e.g., emotional task) [8, 30, 40]. This inconsistency might be because of multiple factors such as different tasks, different signal natures of fMRI and EEG, or different ways to constructing function networks. More high-quality researches are pressed to clarify the consistency.

To address the three questions, we selected the resting-state denoised by FIX (FMRIB's ICA-based X-noiseifier) and task-state (EMOTION and WM) fMRI data from the HCP data with 500 subjects (see Methods for details). Then, we used Pearson's correlation to separately construct three functional networks (FIX, EMOTION, and WM) for each subject. Next, we performed connectivity analysis for EMOTION and WM versus FIX using the NBS toolbox [23] to determine how long connections change during task states versus resting state. We also used the Brain Connectivity Toolbox (BCT) to compute nodal features of participant index (PI) and within-module *Z*-score and global properties (assortativity, global efficiency, and transitivity) and then performed one-way ANOVA with 3 conditions (FIX, EMOTION, and WM) for global and nodal properties at each threshold of 5-15%. Considering that functional networks at task state need to exchange multiple information between different communities, we firstly predicted that although long connections are of a small proportion, they would become more significant relative to resting state because they are responsible for intermodular communication [12, 21]. We also predicted that with the increase of task demands, the number of task-relevant connector and kinless hubs would increase while the number of provincial hubs would decrease [13]. Finally, we predicted that under the disturbance of active tasks, the balance between integration and segregation at resting state would be disrupted and functional networks would tend to be more separated and randomized [8, 30, 40].

## 2. Methods

### 2.1. Participants

After registering an account at ConnectomeDB and agreeing to the Open and Restricted Access Data Use Terms (http://www.humanconnectome.org/), we were approved to download the HCP data with 500 subjects. After matching age with gender and excluding twins, we selected 81 right-handed healthy adults (age 22-30 years old; 40 males and 41 females). These participants had no prior history of neurological or psychiatric disorders.

### 2.2. fMRI Data Selection and Processing

The detailed data acquisition and experimental procedure were described at the HCP website [2]. For structural imaging, T1w was acquired using a 32-channel head coil and 3T Siemens product (MPRAGE and SPACE) sequences (TR = 2400 ms, TE = 2.14 ms, flip angle = 8 degrees, FOV = 224 × 224 mm, voxel size = 0.7 mm isotropic). The selected HCP data included the resting-state and task-state fMRI image datasets. The resting-state fMRI data were acquired in four runs of approximately 15 minutes each, two runs in one session and two in another session, with eyes open with relaxed fixation on a projected bright cross hair on a dark background (and presented in a darkened room) (TR = 720 ms, TE = 33.1 ms, flip angle = 52 degrees, FOV = 208 × 108, matrix = 104 × 90, slice thickness = 2 mm isotropic). Following completion of resting-state fMRI in each of the two fMRI scanning sessions, the task-state data were acquired with the same EPI pulse sequence parameters as the resting-state fMRI. These subject-specific images had been preprocessed through the HCP Minimal Processing Pipelines (MPP): (1) to remove spatial artifacts and distortions; (2) to generate cortical surfaces, segmentations, and myelin maps; (3) to make the data easily viewable in the Connectome Workbench visualization software; (4) to generate precise within-subject cross-modal registrations; (5) to handle surface and volume cross-subject registrations to standard volume and surface spaces; and (6) to make the data available in the CIFTI format in a standard grayordinate space (see [41] for details).

The task-state fMRI data included an emotion processing task and a WM task. The emotion processing task is a Hariri matching task [42], in which the participants were asked to decide which of the two faces presented at the bottom of the screen matched the face at the top of the screen or which of two shapes presented at the bottom of the screen matched the shape at the top of the screen [43]. The faces have either angry or fearful expressions and simple geometric shapes (circles, vertical, and horizontal ellipses) were used as control stimuli. The WM task is an *n*-back task in which 4 different stimulus types (face, places, tools, and body parts) are presented in separate blocks within each run. Within each run, 1/2 of the blocks use a 2-back WM task and 1/2 of the blocks use a 0-back WM task. Each of the two runs contains 8 task blocks (10 trials of 2.5 s each, for 25 s) and 4 fixation blocks (15 s each).

Following Cao et al. [44], the mean average of all task-related signal fluctuations was removed by regression with separate regressors for each experimental condition in order to only account for condition-specific effects, prior to graph construction. The parcellation with 333 parcels developed by Gordon et al. [45] was combined with subcortical areas (bilateral amygdala, hippocampus, accumbens, caudate, pallidum, putamen, thalamus, ventral diencephalon, cerebellum, and the whole brain stem) into a new parcellation with the 352 functional parcels (downloading from https://sites.wustl.edu/petersenschlaggarlab/resources/). Then, we used Connectome Workbench developed by the HCP (http://www.humanconnectome.org/software/connectome-workbench.html) to extract the 352 parcels' time series from the residual task-fMRI data and merged the time series of two scanning orders. Next, we computed the pairwise Pearson's correlation matrices of all these parcel time series for each task. Finally, we removed the rows and columns corresponding to 47 parcels with no original labels in the parcellation developed by Gordon et al. [45] and thus obtained the functional networks with the size 305 × 305.

The resting-state fMRI data contained the FIX data. During the preprocessing, the FIX data had been cleaned of structured noise by a new approach that combines ICA with a more complex automated component classifier referred to as FIX [41]. Similar to the task state, we obtained Pearson's correlation matrices with 305 functional nodes for the FIX data.

### 2.3. Network Connection Analysis

**Figure 1:**
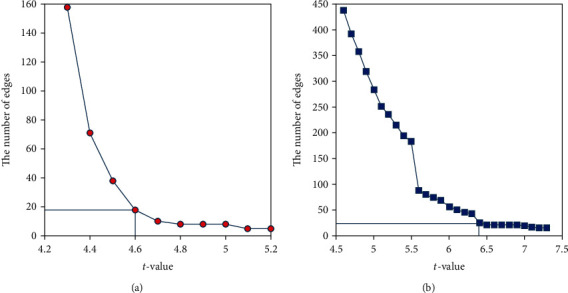
The number of connected edges varies with the threshold of the *t*-value from the network-based statistic method. (a) The inflection point occurs at the threshold of *t* = 4.6 for EMOTION versus resting state. (b) The inflection point occurs at the threshold of *t* = 6.4 for working memory versus resting state.

To identify network connections that varied with the task demand, we used the NBS approach [23]. Full-linking connectivity matrices were entered as repeated measure-dependent variables into the NBS toolbox (freely downloaded from http://www.nitrc.org/projects/nbs/), with the contrast of EMOTION or WM versus FIX. According to Figure 1, the inflection points separately occur at the threshold of *t* = 4.6 for EMOTION versus FIX (i.e., the number of connected edges decreases more sharply when *t* < 4.6; the curve nearly parallels with *t*-axis when *t* > 4.6) and at the threshold of *t* = 6.4 for WM versus FIX (i.e., the curve nearly parallels with *t*-axis when *t* > 6.4). Moreover, the networks for EMOTION and WM versus FIX hold comparable edges at these inflection points. Therefore, an individual-connection-level threshold of *t* = 4.6 and 6.4, respectively, for EMOTION versus FIX and WM versus FIX was used with extent-based correction for multiple comparisons, 5000 permutations, and an overall corrected *p* < 0.0001.

### 2.4. Graph-Theoretic Processing

After these correlation networks were Fisher-Z transformed, their diagonal elements and negative connections were set to zero. We used the BCT (http://www.brain-connectivity-toolbox.net) to sparse functional networks in 1% interval from the threshold 5% to 15%. For each threshold, we constructed weighted networks for the FIX, EMOTION, and WM condition. In these weighted networks, inter/intramodal connections below the threshold were assigned to 0 while the connections above the threshold remained unchanged because weak and nonsignificant links may represent spurious connections that tend to obscure the topology of strong and significant connections and as a result are often discarded [8].

The graph analyses included nodal and global topological features for each threshold. First, to explore how the hubs change in the different task conditions, we computed the nodal PI (or participation coefficients) and within-module degree *Z*-score for each threshold. PI measures the diversity of intermodular connections of individual nodes while within-module degree *Z*-score measures the extent to which a node is connected to other nodes within its module [46]. Following Guimera et al. [27] and Finc et al. [13], we first classified nodes as hubs (*Z*_*i*_ > 1) and nonhubs (*Z*_*i*_ < 1). Then, the hubs were further divided into three classes: (1) provincial hubs with *Z*_*i*_ > 1 and PI_*i*_ < 0.3; (2) connector hubs with *Z*_*i*_ > 1 and 0.3 < PI_*i*_ < 0.75; and (3) kinless hubs with *Z*_*i*_ > 1 and PI_*i*_ > 0.75.

Second, we analyzed the following global network properties to determine functional networks that become integrated or separated from resting to task states. (1) Assortativity is a correlation coefficient between the degrees of all nodes on two opposite ends of a link. A positive assortativity coefficient indicates that nodes tend to link to other nodes with the same or similar degrees. (2) Global efficiency is the inverse of the average shortest path length. (3) Transitivity is the ratio of triangles to triplets in the network and is an alternative to the clustering coefficient.

We wrote the custom Matlab scripts to perform one-way ANOVA with 3 conditions (FIX, EMOTION, and WM) for global and nodal properties separately in weighted networks at each threshold. The Bonferroni method was used for all post hoc analyses. Significant effects of *p* < 0.001 were reported.

## 3. Results

### 3.1. Connectivity-Based Analysis

#### 3.1.1. EMOTION versus FIX Contrast

**Figure 2:**
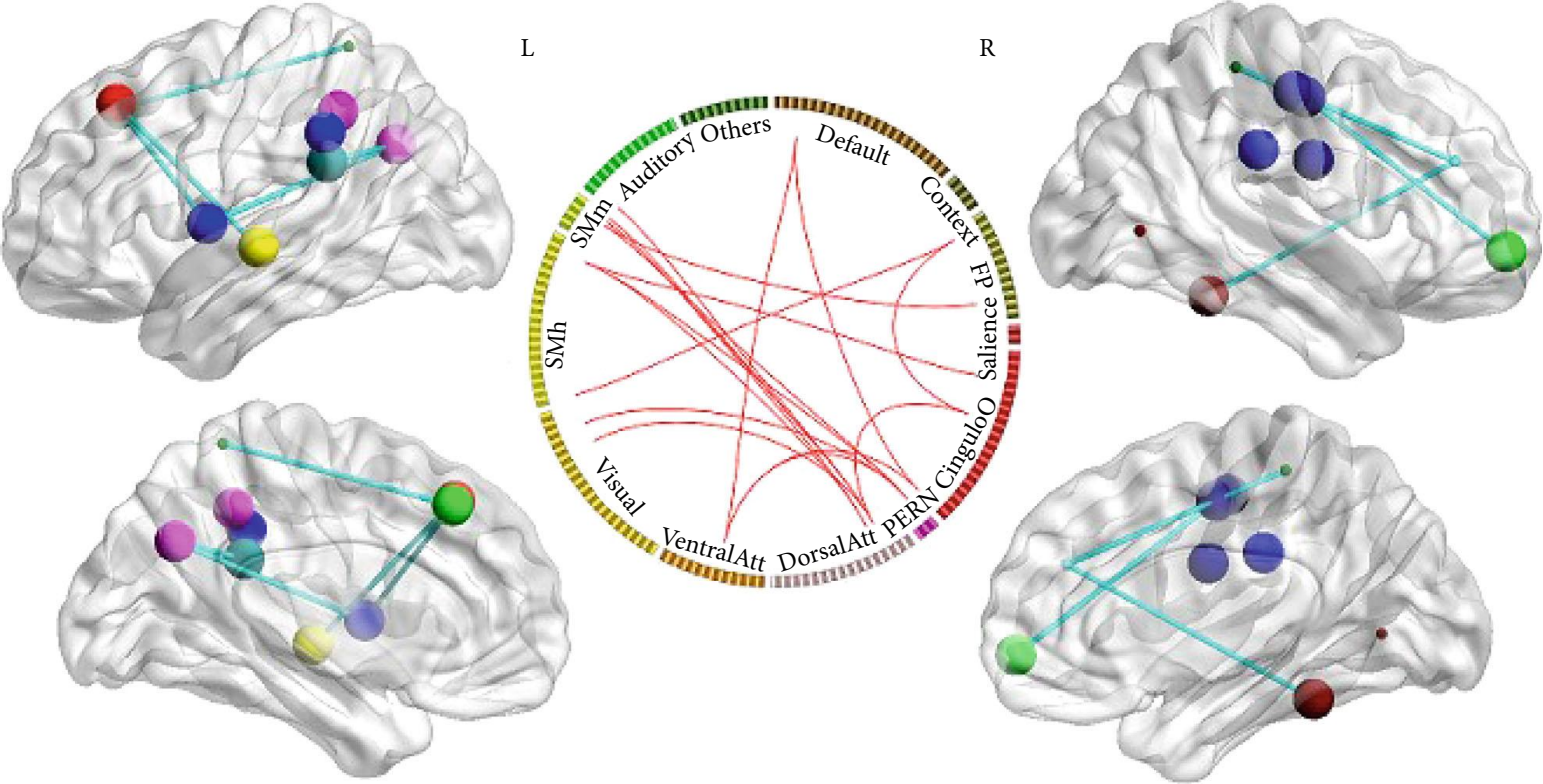
Two-dimension and three-dimension depictions of connectivity analysis result in the EMOTION versus FIX condition. The nodal color denotes affiliative community; the nodal size represents the magnitude of nodal betweenness centrality; the edge depicts binarized edge.

The NBS analysis revealed that a single connected network with 18 nodes and 18 edges was altered (*t* = 4.60, *p* < 0.0001, corrected) (Figure 2).  Table 1 shows the nodes of the connected network. The involved nodal regions included Default_6, two FrontoParietal nodes (3 and 20), three CinguloOperc nodes (7, 17, and 40), PERN_2, two DorsalAttn nodes (6 and 8), one VentralAttn node (11), two Visual nodes (11, 29, and 34), two SMHand nodes (1 and 35), three SMmouth nodes (6, 7, and 8), and one Auditory node (2). All of the connections exhibited increased values in the EMOTION condition compared with the FIX condition. However, there were no significant connections between Context/Salience/Subcortical nodes and other nodes.

#### 3.1.2. WM versus FIX Contrast

**Figure 3:**
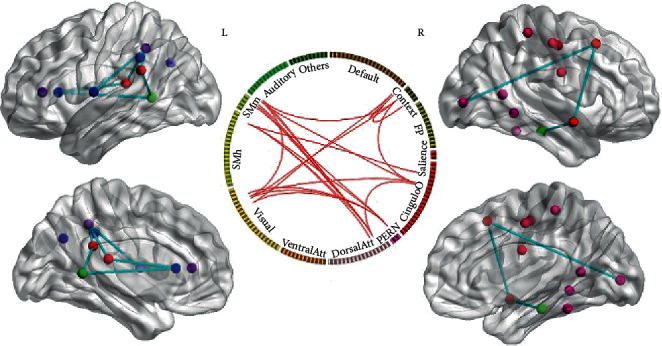
Two-dimension and three-dimension depictions of connectivity analysis result in the WM versus FIX condition. WM: working memory.

The NBS analysis showed a significant increase of connectivity in the WM condition compared with the FIX condition in a single brain network formed by 20 nodes and 23 edges (*t* = 6.40, *p* < 0.0001, corrected) (Figure 3). The involved nodal regions included two Default nodes (40 and 41), two Context nodes (2 and 7), three CinguloOperc nodes (7, 13, and 14), PERN_2, two DorsalAttn nodes (6 and 8), four Visual nodes (28, 31, 32, and 34), SMHand_35, three SMMouth nodes (6, 7, and 8), and two Auditory nodes (1 and 2). There were no significant connections between FrontoParietal/Salience/VentralAttn/Subcortical nodes and other nodes.

### 3.2. Nodal Feature Analysis

**Figure 4:**
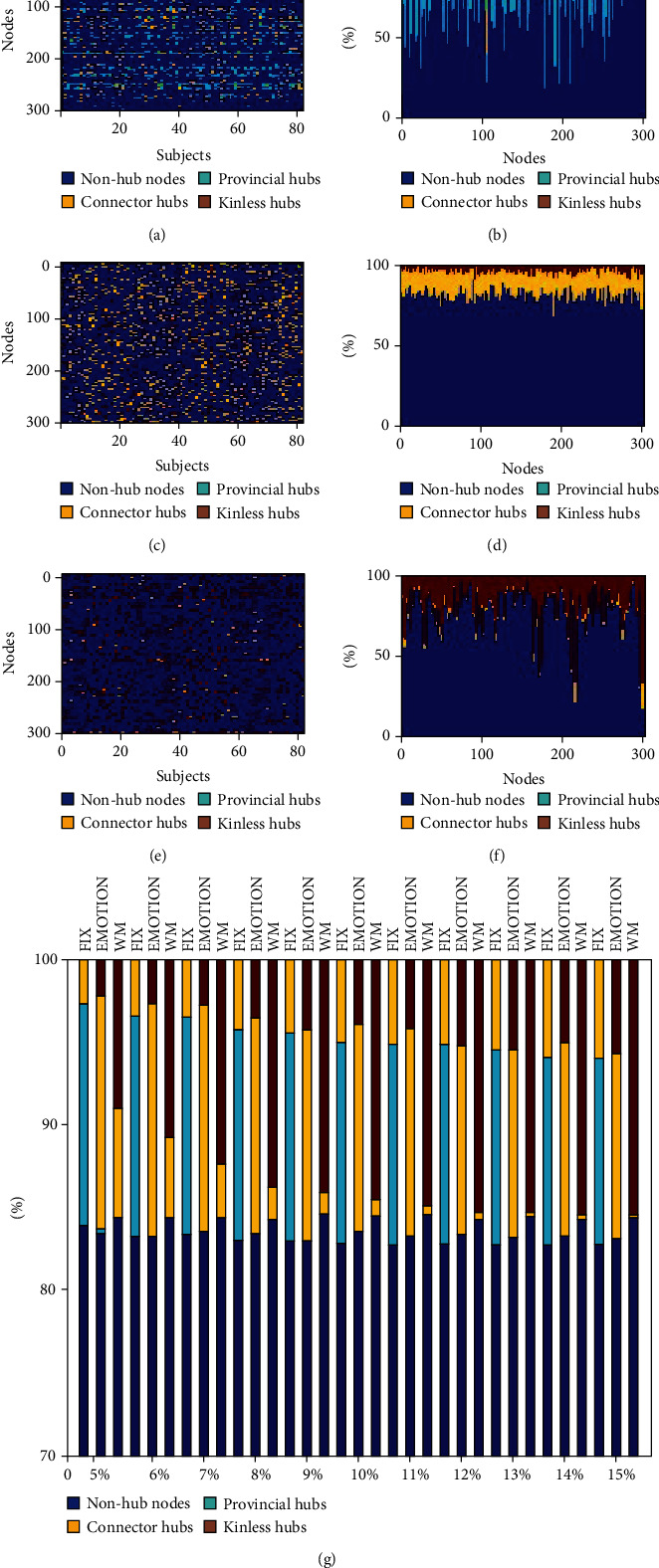
The hub distribution within 305 nodes for 81 subjects. (a, c, and e) Separately describe the hub distribution of FIX, EMOTION, and WM networks at the threshold of 10%. (b, d, and f) Separately describe the subject ratio of each hub in FIX, EMOTION, and WM networks at the threshold of 10%. (g) Describes the mean ratio of the hubs across all 305 nodes and all subjects at the 5-10% threshold.

Figures 4(a)–4(f) show the distribution of the hubs within 305 nodes for 81 subjects at the threshold of 10%, and Figure 4(g) shows the mean ratio of the hubs across all 305 nodes at the 5-10% threshold. Across each threshold, the number of the nonhubs was approximately 83% while the number of the hubs was approximately 17% in the FIX, EMOTION, and WM networks (Figure 4(g)). On the one hand, FIX networks only included provincial (12.42% ± 0.76%) and connector (4.63% ± 1.08%) hubs (Figures 4(a) and 4(b)) but no kinless hubs; EMOTION networks mainly included connector (12.59% ± 1.08%) and kinless hubs (4.11% ± 1.19%) (Figures 4(c) and 4(d)) but no provincial hubs; WM networks mainly covered kinless (13.72% ± 2.15%) and connector (1.90% ± 2.17%) hubs but no provincial hubs (Figures 4(e) and 4(f)). On the other hand, the ratio of the connector hubs increased with thresholds in FIX networks while the ratio of the kinless hubs increased with thresholds in EMOTION and WM networks. Moreover, ANOVA analyses indicated that the ratio of the connector hubs significantly increased in EMOTION networks relative to FIX and WM networks (*p* < 0.001) while the ratio of the kinless hubs significantly increased in WM networks compared to FIX and EMOTION networks (*p* < 0.001).

**Figure 5:**
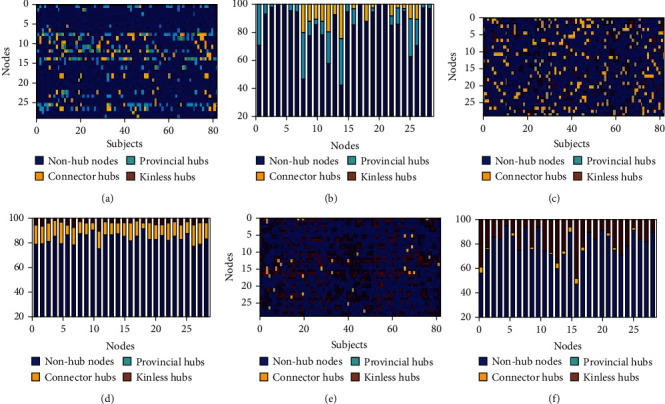
The hub distribution within 28 nodes of FIX (a), EMOTION (c), and WM (e) networks for 81 subjects and the subject ratio of each hub in FIX (b), EMOTION (d), and WM (f) at the threshold of 10%. The nodal order is consistent with Table 1. WM: working memory.

When we ignored the hubs whose subject ratio was less than 5%, we found that the Context, Salience, and Subcortical communities did not include any hubs in the FIX condition. The most nodes of these communities shifted from nonhubs in the FIX condition to connector hubs in the EMOTION condition or to kinless hubs in the WM condition. However, Amygdala_1/2, Putamen_1, and Brain Stem became connector hubs only in the EMOTION condition. Additionally, when we considered the nodes as ROIs in Table 1, we found that Default_41, Context_2/7, FrontoParietal_3/20, Visual_28/31/32/34, SMmouth_6, and Auditory_1/2 in the FIX condition shifted from nonhubs of a single role to connector hubs in the EMOTION condition or to kinless hubs in the WM condition (Figure 5). Similarly, Default_6/40, VentralAttn_11, DorsalAttn_8, and SMhand_35 in the FIX condition switched from provincial hubs of a single role to connector hubs in the EMOTION condition or to kinless hubs in the WM condition. PERN_2 and Visual_29 in the FIX and EMOTION condition changed their single role of connector hubs into kinless hubs in the WM condition. However, CinguloOperc_7/13/14/17/40, DorsalAttn_6, SMhand_1, and SMmouth_7/8 in the FIX condition turned their dual roles of provincial/connector hubs into connector hubs in the EMOTION condition or into kinless hubs in the WM condition.

### 3.3. Global Property Analysis

**Figure 6:**
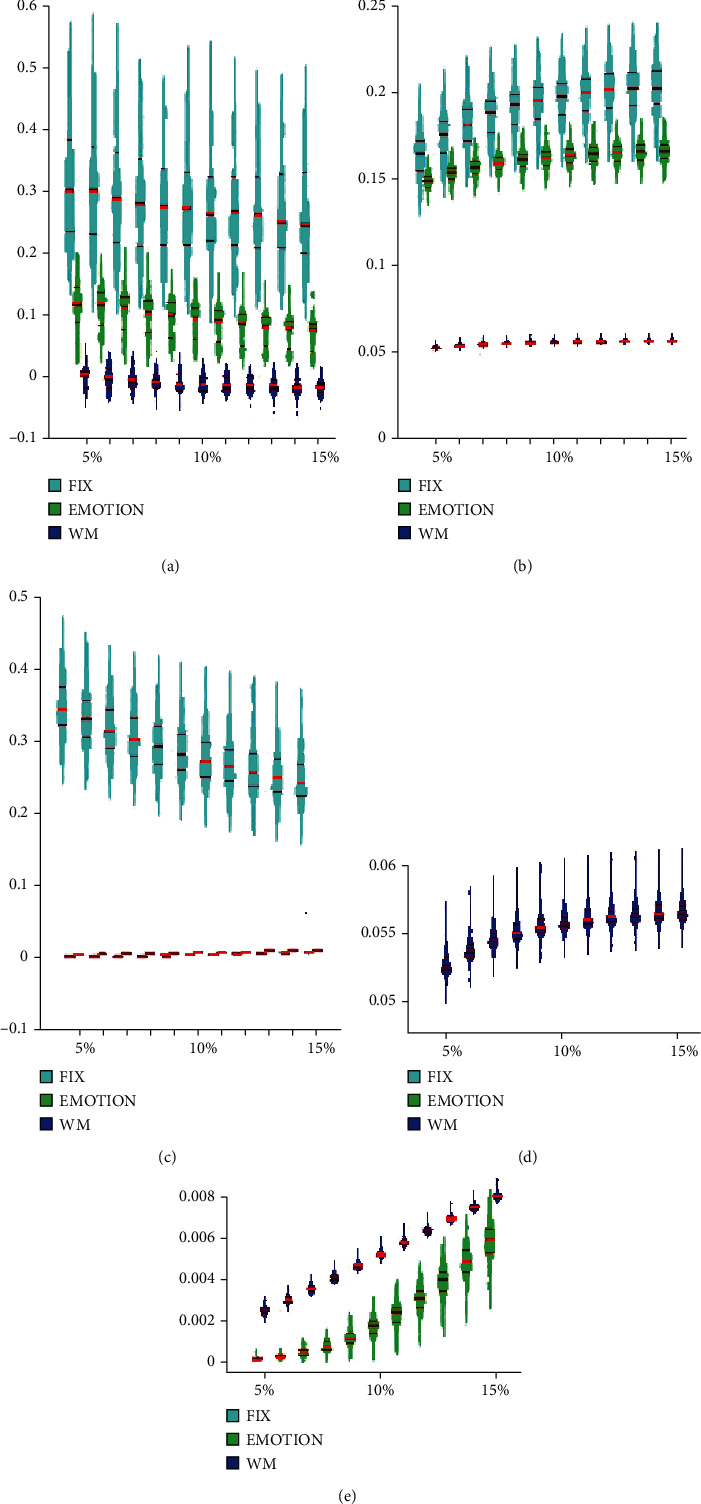
The ANOVA results of global property analysis for FIX, EMOTION, and WM: (a) assortativity; (b) global efficiency; (c) transitivity; (d) global efficiency graph enlarged for WM; (e) transitivity graph enlarged for EMOTION and WM at the threshold of 10%. WM: working memory.

For FIX, EMOTION, and WM, ANOVA analyses on the global network properties showed significant task effects in global efficiency, transitivity, and assortativity (*p* < 0.0001) (Figure 6). Post hoc comparisons indicated that under the threshold of 5-15%, FIX networks contained greater assortativity values than EMOTION networks while EMOTION networks showed greater assortativity values than WM networks (*p* < 0.001) (Figure 6(a)). Moreover, FIX networks exhibited greater global efficiency than EMOTION networks (*p* < 0.001) while EMOTION networks exhibited greater global efficiency than WM networks (*p* < 0.0001) (Figures 6(b) and 6(d)). Finally, FIX networks had greater transitivity values than EMOTION and WM networks (*p* < 0.0001) while WM networks included greater transitivity values than EMOTION networks (*p* < 0.001) (Figures 6(c) and 6(e)).

## 4. Discussion

In the present study, we used graph-theoretic approach to analyze the resting-state and task-state (WM and EMOTION) fMRI scans of 81 subjects from the HCP to determine how the topological properties of functional networks dynamically change according to task demands. Results indicated that relative to resting state, task demands significantly increase the strength of long-distance connections between modules but not within modules; the number of connector and kinless hubs significantly increases in EMOTION and WM networks while provincial hubs disappeared. Moreover, EMOTION and WM networks seem to become separated: their assortativity is close to zero and both the global efficiency and transitivity decreased. These results suggest that task demands change the architecture of intrinsic functional networks and cause local and global topological properties of functional networks at resting state to redistribute.

### 4.1. Long-Distance Connections Dominate Intermodular Communication at Task States

The NBS results indicated that the significant increase in connectivity strength occurred between different communities but not within modules at task state versus resting state. Long-distance connections occupy a relatively small ratio in functional networks [18]. However, a small quantity of long-distance connections is necessary to maintain intermodular information communication because long-distance connection shorten the pathway of information transfer but does not significantly increase the wiring cost [3, 20, 47]. Consist with our results, previous MEEG studies also found that task demands (WM and motor performance) promote synchronization between brain networks through long-distance links [34, 40].

Moreover, WM and EMOTION networks show different connectivity patterns. Particularly, the long connection between Defualt_6 (Frontal_Sup_L) and VentralAttn_11 (Temporal_Mid_L) in EMOTION networks significantly increased while the long connections between Default_40/41 (Frontal_Mid_R and Temporal_Sup_R) and Visual_32/34 (Lingual_R and Fusiform_R) significantly increased in WM networks, which is consistent with the flexible reconfiguration in the interactions of DMN with other subnetworks [19]. However, there were no long connections between Subcortical nodes and other modular nodes. This may be partly attributed to very high threshold during the NBS analysis of fully linking networks. Taken together, long-distance connectivity patterns between modules have decisive significance for decoding multiple task-relevant information.

### 4.2. Connector and Kinless Hubs Dominate Task-State Functional Networks

Consistent with Finc et al. [13], we found that the number of connector hubs that have many links to most of the other modules increased in the EMOTION and WM networks relative to the FIX network. To be noted, kinless hubs that have links homogeneously distributing among all modules also increased, particularly in WM networks. However, kinless hubs did not appear in FIX networks, which might explain why kinless hubs were almost ignored in the literature related to functional networks. In addition, provincial hubs that have the vast majority of links within their own module mainly appeared in FIX networks but disappeared in EMOTION and WM networks. The previous MEG research also found that the motor tapping task causes the shift from resting-state networks dominated by provincial hubs to motor networks with a larger number of connector hubs [38]. Thus, consistent with the flexible hub theory [25], task demands need more between-module information communication so that connector and kinless hubs dominate task-state functional networks.

When we neglected the hubs whose subject ratios were less than 5%, the hubs in EMOTION networks mainly consisted of connector hubs while the hubs in WM networks mainly belonged to kinless hubs. This might be attributed to the fact that PI values in WM networks were higher than those in EMOTION networks. This is also consistent with previous results indicating that more task demands need a more globally synchronized system to involve in [40]. Moreover, bilateral Amygdala, the left Putamen, and Brain Stem became connector hubs only in EMOTION networks, consistent with previous results [20, 26, 28–30], which implies that these hubs take a critical role in decoding emotional information. To be noted, in FIX networks, nonhubs (e.g., Defualt_41, Context_2/7, FrontoParietal_3/20, Visual_28/31/32/34, SMmouth_6, and Auditory_1/2) switch to connector hubs in EMOTION networks or kinless hubs in WM networks. These results suggest that task-relevant functional nodes dynamically reconfigure and shift their network roles from within-module to between-module.

### 4.3. Functional Networks at Task State Tend to Be More Separated Than Those at Resting State

Intrinsic functional networks at resting state represent a standard architecture and maintain the balance between integration and separation, which is evoked by task-relevant network changes [6, 14]. Although less than 24 long-distance connections appeared and less than 17% functional nodes switched their roles in EMOTION and WM as discussed above, the global properties changed significantly. The global efficiency and transitivity significantly decreased in EMOTION and WM versus FIX, which means that task demands cause increase in pathway lengths (e.g., long connections appear) and decrease in clustering coefficients. This is inconsistent with increases in task demands leading to more integrated brain networks [36, 37]. The previous study used a binary network to compute global properties [36] while our study used weighted networks. Undoubtedly, weighted correlation networks occupied more accurate representation than the binary networks.

What is more, the assortativity values reflecting a correlation coefficient between the degrees of all nodes significantly decreased and were close to zero at task state. Similarly, previous results also found that affective networks have negative assortativity and lower global efficiency and exhibit weaker small-worldness [30]. These results suggest that task demands break the balance between local and distant functional couplings at resting state [16] and cause functional networks to reconfigure their topologies. As a result, functional networks at task state tend to become more separated or random, a shift of network architecture to a more random configuration at higher levels of task difficulty [8, 30, 40]. Our result showing that more kinless hubs appeared in WM than in EMOTION networks to some degree provide direct evidence for this opinion. However, functional brain network topology was never completely randomized because of the constraint of structural network [8]. These explanations are not in agreement with previous MEG/EEG results that the clustering coefficient was conserved over a wide range of frequencies and increasing memory load increased clustering coefficient [38, 39]. One possibility is because of the difference in signal measurement nature between fMRI and MEEG. Another possible interpretation is because the previous research absolutized the correlation between wavelet coefficients for each pair of sensors [38] or used EEG phase synchronization (positive and negative value) as a functional connectivity index [39], while the present study only contained positive connections. To clarify these inconsistencies, future studies necessarily combine fMRI with MEEG and select hard- and soft-thresholding approach of functional networks [48].

In summary, task demands break the balance between local and global coupling among brain regions in intrinsic functional networks. Long-distance functional connections dominated intermodular communication of functional networks at task states under high threshold. Correspondently, task-relevant connector or kinless hubs between modules were flexibly redistributed to promote task performance while provincial hubs within modules disappeared. Moreover, task-state networks significantly change their global topologies and tend to become more random. These observations provide important and direct evidences for the understanding of the organization principle behind the functional architecture of task-state networks. To be cautious, it is unclear whether our results can be generalized to other task-state network. Future studies should use more other tasks with other network properties to further confirm our observations.

## Figures and Tables

**Table 1:** The nodes of connected network in the emotion and working-memory versus the resting-state condition during the network-based statistical analysis.

ID	Parcel label	AAL	Centroid MNI			Role^∗^
			*x*	*y*	*z*	
6	Default_6	Frontal_Sup_L	-19.5	30.1	45.5	P
40	Default_40	Frontal_Mid_R	30.6	18.9	48.7	P
41	Default_41	Temporal_Sup_R	54.4	1.1	-12.9	Nonhub
43	Context_2	Calcarine_L	-8.8	-49.8	4.2	Nonhub
48	Context_7	Fusiform_R	34.6	-23.9	-20.4	Nonhub
52	FrontoParietal_3	Frontal_Sup_Media	-5.5	29.3	44	Nonhub
69	FrontoParietal_20	Frontal_Mid_Orb_R	28.4	57	-5.1	Nonhub
84	CinguloOperc_7	SupraMarginal_L	-57.7	-40.6	35.8	P/C
90	CinguloOperc_13	Insula_L	-28.8	23.7	8.4	P/C
91	CinguloOperc_14	Rolandic_Oper_L	-59.8	-4.1	8.8	P/C
94	CinguloOperc_17	Rolandic_Oper_L	-51.8	-0.6	5	P/C
117	CinguloOperc_40	SupraMarginal_R	54.9	-27	29.6	P/C
119	PERN_2	Precuneus_L	-12.7	-64.9	31.8	C
128	DorsalAttn_6	Parietal_Inf_L	-42.9	-45	43	P/C
130	DorsalAttn_8	Frontal_Inf_Tri_L	-43.6	36.3	8.5	P
165	VentralAttn_11	Temporal_Mid_L	-59	-18	-3	P
205	Visual_28	Occipital_Mid_R	31.7	-85.7	2.4	Nonhub
206	Visual_29	Lingual_R	43.8	-67.2	2	C
208	Visual_31	Temporal_Mid_R	49	-54.5	8.8	Nonhub
209	Visual_32	Lingual_R	31.2	-45.6	-5.8	Nonhub
211	Visual_34	Fusiform_R	34.9	-44	-20	Nonhub
217	SMhand_1	Cuneus_R	-18.8	-48.7	65	P/C
251	SMhand_35	Postcentral_R	39.2	-34.6	57.5	P
260	SMmouth_6	Precentral_R	42.3	-11	47.3	Nonhub
261	SMmouth_7	Postcentral_R	53.9	-8.3	26.1	P/C
262	SMmouth_8	Precentral_R	47.8	-15.1	49.3	P/C
263	Auditory_1	Heschl_L	-32	-29.3	15.6	Nonhub
264	Auditory_2	SupraMarginal_L	-46.3	-41.4	25.9	Nonhub

Note. P: provincial hub; C: connector hub. ^∗^Nodal role at the resting state.

## Data Availability

The data that support the findings of this study are available from the corresponding author upon reasonable request.
